# Body mass index and waist circumference combined predicts obesity-related hypertension better than either alone in a rural Chinese population

**DOI:** 10.1038/srep31935

**Published:** 2016-08-22

**Authors:** Ming Zhang, Yang Zhao, Guoan Wang, Hongyan Zhang, Yongcheng Ren, Bingyuan Wang, Lu Zhang, Xiangyu Yang, Chengyi Han, Chao Pang, Lei Yin, Jingzhi Zhao, Dongsheng Hu

**Affiliations:** 1 Department of Preventive Medicine, Shenzhen University Health Sciences Center, Shenzhen, Guangdong, People’s Republic of China; 2 The Affiliated Luohu Hospital of Shenzhen University Health Sciences Center, Shenzhen, Guangdong, People’s Republic of China; 3 Department of Epidemiology and Health Statistics, College of Public Health, Zhengzhou University, Zhengzhou, Henan, People’s Republic of China; 4 Department of Pharmacology, Shenzhen University Health Sciences Center, Shenzhen, Guangdong, People’s Republic of China; 5 Department of Prevention and Health Care, Military Hospital of Henan Province, Zhengzhou, Henan, People’s Republic of China

## Abstract

Limited information is available on the association of obesity defined by both body mass index (BMI) and waist circumference (WC) with incident hypertension in rural China. A total of 9,174 participants ≥18 years old from rural areas in middle of China, free of hypertension, diabetes, myocardial infarction and stroke, were selected in this cohort study. Questionnaire interview and anthropometric and laboratory measurements were performed at baseline (2007–2008) and follow-up (2013–2014). During the 6 years of follow-up, hypertension developed in 733/3,620 men and 1,051/5,554 women. After controlling for age, education level, smoking, drinking, physical activity, and family history of hypertension, the relative risk of hypertension was lower for participants with high BMI but normal WC than those with both BMI and WC obesity for men 18–39 and 40–59 years old. Women 18–39 years old with normal BMI but high WC showed a 1.96-fold risk of hypertension, and being female with age 40–59 years and high BMI but normal WC was independently associated with hypertension incidence as compared with both normal BMI and WC. BMI is more associated with hypertension as compared with WC in both genders. High WC tends to add additional risk of hypertension in young women.

In 2010, there were an estimated 265 million hypertensive patients (20–79 years) in China, making this country the largest absolute disease burden of hypertension in the world^1^. Hypertension is an important public health challenge in China and measures are required at a population level to prevent the development of hypertension^2^.

Obesity is associated with increased prevalence of hypertension^3^. Anthropometric indices including body mass index (BMI) and waist circumference (WC) are used most frequently to define different obesity categories among various populations^4^. Presently, general obesity classified by BMI and central obesity classified by WC are both confirmed to be associated with incident hypertension in Chinese adults^5^,^6^. Studies often investigated general and central obesity separately, however, not all people with obesity have both high BMI and WC. A cohort study of US adults found that increased WC may not be related to the change in BMI and suggested that a combination of BMI and WC may provide a better prediction of obesity-related disease than sole using of BMI or WC alone^7^. Subsequently, Du T *et al.* indicated that approximately two thirds of obese people would be missed if WC were not measured in China^8^. These findings implied the importance and necessity of identifying the specific obesity categories defined by BMI and WC simultaneously for predicting obesity-related hypertension.

Nowadays, the prevalence of obesity is showing a remarkable increasing upwards trend in Chinese rural areas^9^. However, little research has investigated people living in rural areas^10^,^11^. Whether people with normal BMI but high WC or high BMI but normal WC would show increased risk of hypertension is unclear. Therefore, the aim of our present study was to describe the prevalence of obesity according to both BMI and WC levels, and investigate the relationship between various obesity categories and the risk of hypertension among people ≥18 years old in rural China.

## Results

### Demographic and clinical characteristics of study participants

A total of 2,727 participants who met the inclusion criteria at baseline, however, refused to participate in follow-up examination. The comparison of baseline characteristics between participated and non-participated study population are shown in Table 1. Compared with the group of non-participated, the group of participated was older, showed lower proportion of men, high school graduation, and drinking, and had higher levels of BMI, WC, GLU, TC, TG, and LDL (all *p* < 0.001).

Baseline characteristics of the study participants by blood pressure status at follow-up are in Table 2. Median (range) of age for normotensive and hypertensive groups was 45.00 (38.00–55.00) and 52.00 (43.00–61.00) years, respectively (*p* < 0.001). Compared with normotensive participants, those with hypertension had positive family history of hypertension (33.9% vs. 29.1%, *p* < 0.001) and higher median BMI and WC (both *p* < 0.001). The prevalence of BOWO for normotensive and hypertensive participants was 30.2% and 42.2%, respectively (*p* < 0.001), with no significant difference in prevalence of BOWN and BNWO between the two groups. Levels of GLU, TC, TG, and LDL were significantly higher in hypertensive than normotensive participants (*p* < 0.001 for all levels), whereas HDL was higher for normotensive participants (*p* < 0.001).

### Prevalence of various obesity categories

The prevalence of central obesity was higher than general obesity for each age group for both men and women (all *p* < 0.05) (Table 3). Also, women tended to have higher age-specific prevalence of central obesity as compared with men (all *p* < 0.05). Men 40 to 59 years old showed the highest prevalence of central obesity (23.1%), BOWN (20.0%), BNWO (0.9%), and BOWO (22.1%), except for general obesity (8.1%). Men ≥60 years old showed the lowest prevalence of general and central obesity, BOWN, and BOWO (all *p* < 0.05). Among each age group, men showed greater prevalence of BOWN than BNWO (all *p* < 0.05).

Women ≥60 years old had the highest prevalence of central obesity (54.2%) and BNWO (17.4%) and the prevalence was higher for BNWO than BOWN (17.4% vs. 5.1%, *p* < 0.001); however, the prevalence was higher for BOWN than BNWO for women 18 to 39 years old (9.0% vs. 6.4%, *p* = 0.006).

### Incident hypertension among rural Chinese

**Figure 1:**
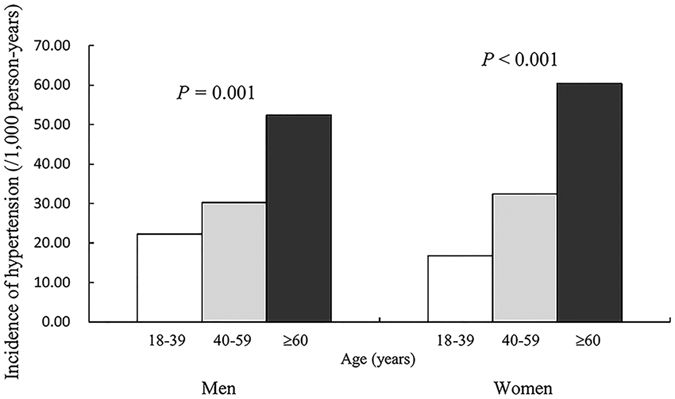
Incidence of hypertension in rural Chinese adults ≥18 years old by gender and age groups.

**Figure 2:**
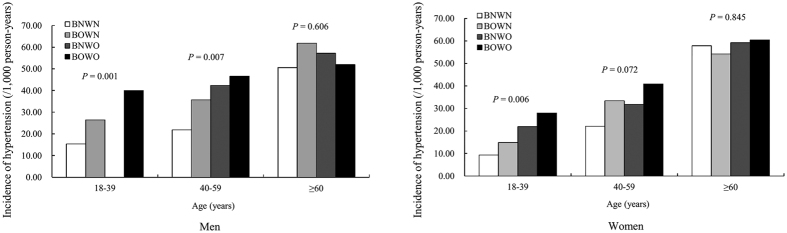
Incidence of hypertension in rural Chinese adults ≥18 years old by obesity and age groups. BNWN, BOWN, BNWO, and BOWO defined using BMI (kg/m^2^) and WC (cm) levels. BNWN, 18.5≤BMI<24, WC<90 for men and WC<80 for women; BOWN, BMI≥24, WC<90 for men and WC<80 for women; BNWO, 18.5≤BMI<24, WC≥90 for men and WC≥80 for women; BOWO, BMI≥24, WC≥90 for men and WC≥80 for women.

During the 6-year follow-up, the overall cumulative incidence of hypertension was 20.25% (733 cases, 33.21/1,000 person-years) for men and 18.92% (1,051 cases, 31.53/1,000 person-years) for women. Overall, among all hypertensive participants, only 48.5% (47.7% for men and 49.9% for women) were aware of their condition, 47.1% (44.1% for men and 49.2% for women) were taking anti-hypertensive medication to lower their blood pressure, and 26.6% (24.6% for men and 28.1% for women) achieved blood pressure control (Table 1 in the Supplement). The incidence of hypertension increased with increasing age for both genders (both *p* < 0.05) (Fig. 1). The incidence for men aged 18–39, 40–59 and ≥60 years was 22.30, 30.23, and 52.44/1,000 person-years, respectively, and for women was 16.77, 32.45, and 60.39/1,000 person-years, respectively. Among men aged 40–59 years, the incidence of hypertension for BNWN, BOWN, BNWO, and BOWO groups was 21.83, 35.69, 42.33, and 46.58/1,000 person-years, respectively, and among women 18-39 years was 9.31, 14.86, 21.92, and 27.97/1,000 person-years, respectively (Fig. 2).

### Multivariate risk assessment

For both genders of participants aged 18–39 and 40–59 years, with obesity defined by BMI or WC, the risk of developing hypertension was increased (Table 4). For men 18–39 years old, the adjusted HRs (95% CIs) for developing hypertension with BOWN and BOWO was 1.78 (1.06–2.99) and 2.29 (1.45–3.62), respectively, and for those 40–59 years old was 2.02 (1.53–2.68) and 2.32 (1.79–3.00), respectively, as compared with BNWN. Being female with BNWO and 18–39 years old was independently related to incidence of hypertension (adjusted HR = 1.96, 95% CI = 1.07–3.58), as was being female with BOWN and 40–59 years old (adjusted HR = 1.57, 95% CI = 1.14–2.16), as compared with BNWN. Women with BOWO showed the highest risk of hypertension for those 18–39 (adjusted HR = 2.81, 95% CI = 1.96–4.03) and 40–59 (adjusted HR = 1.77, 95% CI = 1.45–2.16) years old as compared with BNWN participants. None of the obesity categories were potential risk factors of hypertension for either gender ≥60 years old.

## Discussion

In this large prospective study, obesity defined by BMI or WC was positively and significantly associated with increased risk of incident hypertension except for participants ≥60 years old. Compared with non-obese participants, men 18–59 years old and women 40–59 years old with high BMI but normal WC still showed increased risk of developing hypertension; furthermore, the hypertension risk was increased for women 18–39 years old with normal BMI but high WC.

A nationwide survey of China reported that hypertension is highly prevalent among Chinese population (28.9% for men and 24.5% for women) in 2010.(ref. 1) The rates of awareness, treatment, and control for hypertension among hypertensive patients were 30.6%, 24.7%, and 6.1% respectively, in 2002, and increased greatly to 44.6%, 35.2% and 11.2%, respectively, in 2010 1,12. Data from the China Health and Nutrition Survey suggested that the incidence of hypertension in 1991–1997, 1993–2000, 1997–2004, and 2000–2006 were 34, 32, 35, and 38/1,000 person-years, respectively, for men, and 26, 26, 27, and 28/1,000 person-years, respectively, for women^13^. Increased age and BMI, physical inactivity, and living in rural region contributed the increased trend in incidence of hypertension in China^13^. The age-adjusted prevalence rates of awareness (37.3%), treatment (27.6%), and control (6.5%) for hypertension were inadequate in rural China in 2010, which calling for urgent improvements in hypertension prevention and control programs among the rural Chinese people^1^.

We found significant differences by gender in prevalence of central obesity. Women tended to have high WC as compared with men. These findings are consistent with a previous study performed in China^14^. Furthermore, our data showed that men had greater age-specific prevalence of BOWN but lower of BNWO. In contrast, women had similar prevalence of BOWN and BNWO. The explanation for this finding might be the difference in body composition by gender. Men were likely to have a greater skeletal muscle than women and women tend to have greater percentage of body fat than men, confirmed in previous studies^15^. Our results also showed that 48.5% of men and 30.3% of women showed single BMI obesity or WC obesity among the obese population. These data indicate the need to use BMI and WC simultaneously to better identify obese individuals by gender.

Controversy remains regarding whether BMI than WC is more closely associated with hypertension. Evidence from previous study demonstrated that BMI and WC were equally well related to hypertension in all participants regardless of age and gender^4^. However, some studies indicated that central obesity may be a better predictor for the risk of hypertension and other cardiovascular diseases^16^,^17^. A cross-sectional Chinese study of 500,000 adults indicated that general rather than central obesity was more strongly associated with high blood pressure^18^. Likewise, our results showed that both general and central obesity were positively and significantly associated with increased risk of incident hypertension except for participants ≥60 years old. In reality, the blood pressure of adults increases with age^19^. The incidence of hypertension increased rapidly with age in men and women, which was also observed in the previous population-based studies in Chinese and other populations^20^,^21^. Hence, the elderly should monitor their blood pressure regularly to identify the occurrence of hypertension in time regardless of their obese status. Optimal body weight control and reduced central obesity risk may have beneficial effects on hypertension control, especially for young and middle-aged adults.

In this study, participants with high BMI but normal WC had elevated risk of developing hypertension for men 18–59 years old and women 40–59 years old, respectively, as compared with BNWN participants. Our results, together with previous study, provide evidence that BMI is related more to hypertension than WC, and people should take part in weight control programs to keep BMI at a moderate level^10^. A Chinese cross-sectional study suggested that adults with normal BMI but high WC had a 1.96-fold risk of hypertension^9^. Our data showed that the incidence of hypertension was higher for BNWO than BNWN participants only for women 18–39 years old; furthermore, those with BNWO also showed a 1.96-fold increased risk of developing hypertension after adjusting for potential confounding variables. WC may add an additional risk of hypertension particularly in young women with normal BMI. According to these differences by age and gender, the ability of BMI and WC to predict obesity-related disease risk may vary in different populations. Whether WC is normal or not, high BMI is significantly associated with hypertension in young and middle aged men. Young women should observe changes in visceral adipose distribution closely and keep a healthy BMI and WC.

Our study was a well-designed large prospective cohort study: BMI and WC were combined to create another 3 obesity groups to evaluate the relationship between obesity and hypertension risk and better identify all the subgroups of obese people at high risk of hypertension. However it has several limitations. First, the study sample was from one county in the middle area of China, which is not a representative sample of the Chinese rural population. Second, we focused on obesity at baseline. Duration of obesity and obese status at follow-up visit were not analyzed in current study. Third, our questionnaire did not contain psychosocial questions. Information on psychological illness such as stress or depression were not available which are associated with cardiovascular health in Chinese China, especially among rural population^22^.

In conclusion, our study provides longitudinal evidence for the association of obesity and incident hypertension among rural Chinese population. Both general and central obesity were associated with increased risk of hypertension. Young and middle-aged men with a small increase in BMI, although WC in the normal range, carried an excess risk of hypertension. Even a modest gain in WC with weight at a normal level was associated with a substantially higher risk of hypertension in young women. Early prevention and control of obesity and hypertension are regarded as a cost-effective approach to decrease economic and health burdens of chronic non-communicable diseases worldwide^23^. Weight-loss programs should be focused as essential elements in the future hypertension prevention and control systems in rural China. Young women should pay more attention to changes in visceral adipose distribution and keep both BMI and WC values in normal ranges to reduce the risk of hypertension.

## Methods

### Study population

This cohort study was conducted in the rural area in Xin’an, a county of Luoyang City in the middle of China^24^,^25^. At baseline, a total of 20,194 participants ≥18 years old without severe psychological disorders, physical disabilities, Alzheimer’s disease, dementia, tuberculosis, Acquired Immune Deficiency Syndrome, or other infectious diseases were recruited for this study during July to August 2007 and July to August 2008. Detailed descriptions of the study design and study population have been published previously^26^,^27^. We excluded participants with hypertension (n = 6,299), myocardial infarction (n = 89), stroke (n = 127), or diabetes (n = 830) at baseline; underweight defined by BMI ≤8.5 kg/m^2^ (n = 580) at baseline; of missing indicators of BMI and WC (n = 6) at baseline. We excluded data for participants who died before the follow-up examination (n = 362) or those who refused to participate in the follow-up examination (n = 2,727). Participants with myocardial infarction, stroke and diabetes at baseline were excluded because these disorders can directly affect blood pressure; moreover, these patients tended to be on medications that could further decrease blood pressure. Ultimately, data for 9,174 eligible participants were included in this analysis. The study was conducted in accordance with the Declaration of Helsinki and approved by the Ethics Committee of Shenzhen University Health Sciences Center. Written informed consent was obtained from all participants before any sample or data collection.

### Baseline measurements

Data were collected on sociodemographic characteristics, behavioral measures, dietary characteristics and personal and family histories of diseases by a questionnaire administered during face-to-face interviews by trained interviewers. The description of the questionnaire was previously published^24^.

Body weight, height, and WC were measured twice with a metric scale and a vertical weight scale with a standardized protocol^28^. Body weight and height were measured to the nearest 0.5 kg and 0.1 cm, respectively, with participants wearing light clothes and in bare feet. WC was measured at the mid-point between the lowest rib and the iliac crest to the nearest 0.1 cm with participants gently breathing. BMI was calculated as weight (kg) divided by height (m) squared.

Blood pressure was measured three times in the right arm, at an interval of 30 seconds, by using an electronic sphygmomanometer (HEM-770AFuzzy, Omron, Kyoto, Japan) during one visit. Participants were instructed refrain from consuming cigarettes, alcohol, coffee and tea and from performing exercise for at least 5 min before blood pressure measurement and were seated comfortably with the arm positioned at the level of the heart during the measurement. The average of three measurements was considered as blood pressure value.

An overnight fasting blood sample from each participant was collected into a vacuum tube for assessing serum levels of glucose (GLU), total cholesterol (TC), triglycerides (TG), and high density lipoprotein (HDL). The concentration of low density lipoprotein (LDL) was calculated by the Friedewald formula^29^. Details about the storage and measurement methods were published previously^24^.

### Follow-up measurements

Follow-up data were collected during July to August of 2013 and July to October of 2014 by questionnaire interview, physical examination, and laboratory measurements as for the baseline examination. Incident cases of hypertension were ascertained from blood pressure value and use of anti-hypertensive medication at the follow-up examination.

### Definition of hypertension and obesity

According to National High Blood Pressure Education Program, hypertension was defined as systolic blood pressure (SBP)≥140 mmHg or diastolic blood pressure (DBP)≥90 mmHg, and currently using of anti-hypertension medication^30^.

The Chinese standard of general obesity was defined by BMI based on the International Life Science Association: 18.5 kg/m^2^≤BMI<24 kg/m^2^ for normal weight; 24 kg/m^2^≤BMI<28 kg/m^2^ for overweight; BMI≥28 kg/m^2^ for general obesity^31^. Central obesity was defined by WC according to the International Diabetes Federation Epidemiology Task Force Consensus Group: WC≥90 cm for men and WC≥80 cm for women according to the recommended criteria for Chinese adults^32^. Except for general and central obesity, BMI and WC were combined to create four new obesity categories as follows: both BMI and WC normal (BNWN): 18.5 kg/m^2^≤BMI<24 kg/m^2^, WC<90 cm for men and WC<80 cm for women; BMI obesity and WC normal (BOWN): BMI≥24 kg/m^2^, WC<90 cm for men and WC<80 cm for women; BMI normal and WC obesity (BNWO): 18.5 kg/m^2^≤BMI<24 kg/m^2^, WC≥90 cm for men and WC≥80 cm for women; both BMI and WC obesity (BOWO): BMI≥24 kg/m^2^, WC≥90 cm for men and WC≥80 cm for women.

### Statistical analysis

Numerical variables (not normally distributed) are presented as median and range and categorical variables as percentage, as appropriate, by blood pressure status at follow-up. Mann-Whitney U test was used to compare the median values of baseline risk factors and χ^*2*^ test was used to compare the proportions between normotensive and hypertensive participants. Differences in the prevalence of obesity categories and incidence of hypertension among age groups were estimated by χ^*2*^ test. The Incidence of hypertension by gender and age was calculated by person-years of follow-up (per 1,000 person-years), with differences tested using χ^*2*^ test. Hazard ratios (HRs) and 95% confidence intervals (CIs) for different obesity measurements associated with hypertension were calculated by Cox proportional hazard model with adjustment for age, education level, smoking, drinking, physical activity, and family history of hypertension. All the analyses involved use of SPSS 21.0 (SPSS Inc., Chicago, IL, USA) and all reported *p*-values were two-sided, with *p* < *0.05* considered statistically significant.

## Additional Information

**How to cite this article**: Zhang, M. *et al.* Body mass index and waist circumference combined predicts obesity-related hypertension better than either alone in a rural Chinese population. *Sci. Rep.* **6**, 31935; doi: 10.1038/srep31935 (2016).

## Supplementary Material

Supplementary Information

## Figures and Tables

**Table 1:** Comparison of baseline characteristics of participants and non-participants.

Variables	Participants (n = 9174)	Non-participants (n = 2727)	*p* value
Men	3620 (39.5)	1192 (43.7)	<0.001
Age (years)	47.00 (39.00–56.00)	40.00 (32.00–51.00)	<0.001
Education level			
High school or higher education level	1021 (11.1)	587 (21.6)	<0.001
Smoking	2597 (28.3)	782 (28.7)	0.709
Drinking	1191 (13.0)	449 (16.5)	<0.001
Physical activity			<0.001
Low	4876 (53.2)	1115 (40.9)	
Moderate	1989 (21.7)	649 (23.8)	
High	2309 (25.2)	963 (35.3)	
Family history of hypertension	2756 (30.0)	846 (31.0)	0.327
BMI (kg/m^2^)	23.58 (21.51–25.84)	23.08 (21.01–25.32)	<0.001
WC (cm)	80.25 (74.00–87.00)	78.25 (72.50–86.25)	<0.001
GLU (mmol/L)	5.26 (4.94–5.62)	5.22 (4.92–5.54)	<0.001
TC (mmol/L)	4.26 (3.73–4.89)	4.10 (3.57–4.71)	<0.001
TG (mmol/L)	1.28 (0.91–1.82)	1.19 (0.85–1.72)	<0.001
HDL (mmol/L)	1.14 (0.99–1.32)	1.14 (0.98–1.32)	0.628
LDL (mmol/L)	2.50 (2.00–2.90)	2.30 (1.90–2.80)	<0.001
BNWN*	4519 (49.3)	1564 (57.4)	<0.001
BOWN*	1151 (12.5)	351 (12.9)	0.654
BNWO*	520 (5.7)	108 (4.0)	<0.001
BOWO*	2984 (32.5)	704 (25.8)	<0.001
General obesity	948 (10.3)	269 (9.9)	0.478
Central obesity	3504 (38.2)	812 (29.8)	<0.001

BMI, body mass index; WC, waist circumference; GLU, glucose; TC, total cholesterol; TG, triglycerides; HDL, high density lipoprotein; LDL, low density lipoprotein.

Data are number (percentage) or median (range).

p value for comparison between participants and non-participants.

^*^BNWN, BOWN, BNWO, and BOWO defined using BMI (kg/m2) and WC (cm) levels. BNWN, 18.5≤BMI<24, WC<90 for men and WC<80 for women; BOWN, BMI≥24, WC<90 for men and WC<80 for women; BNWO, 18.5≤BMI<24, WC≥90 for men and WC≥80 for women; BOWO, BMI≥24, WC≥90 for men and WC≥80 for women.

**Table 2:** Baseline characteristics of rural Chinese adults ≥18 years old by blood pressure status at follow-up.

Variables	Normotension (n = 7390)	Hypertension (n = 1784)	*p* value
Men	2887 (39.1)	733 (41.1)	0.117
Age (years)	45.00 (38.00–55.00)	52.00 (43.00–61.00)	<0.001
Education level			
High school or higher education level	851 (11.5)	170 (9.6)	0.135
Smoking	2090 (28.3)	507 (28.4)	0.908
Drinking	970 (13.1)	221 (12.4)	0.405
Physical activity			
Low	3915 (53.0)	961 (53.9)	0.066
Moderate	1637 (22.2)	352 (19.7)	
High	1838 (24.9)	471 (26.4)	
Family history of hypertension	2152 (29.1)	604 (33.9)	<0.001
BMI (kg/m^2^)	23.36 (21.36–25.62)	24.42 (22.37–26.63)	<0.001
WC (cm)	79.65 (73.50–86.25)	83.25 (76.75–89.75)	<0.001
GLU (mmol/L)	5.24 (4.92–5.59)	5.37 (5.04–5.74)	<0.001
TC (mmol/L)	4.22 (3.70–4.85)	4.41 (3.88–5.05)	<0.001
TG (mmol/L)	1.25 (0.90–1.78)	1.40 (1.00–1.98)	<0.001
HDL (mmol/L)	1.15 (0.99–1.33)	1.13 (0.97–1.30)	<0.001
LDL (mmol/L)	2.40 (2.00–2.90)	2.60 (2.10–3.00)	<0.001
BNWN*	3842 (52.0)	677 (37.9)	<0.001
BOWN*	913 (12.4)	238 (13.3)	0.259
BNWO*	403 (5.5)	117 (6.6)	0.070
BOWO*	2232 (30.2)	752 (42.2)	<0.001
General obesity	696 (9.4)	252 (14.1)	<0.001
Central obesity	2635 (35.7)	869 (48.7)	<0.001

BMI, body mass index; WC, waist circumference; GLU, glucose; TC, total cholesterol; TG, triglycerides; HDL, high density lipoprotein; LDL, low density lipoprotein.

Data are number (percentage) or median (range).

p value for comparison between normotension and hypertension.

^*^BNWN, BOWN, BNWO, and BOWO defined using BMI (kg/m2) and WC (cm) levels. BNWN, 18.5≤BMI<24, WC<90 for men and WC<80 for women; BOWN, BMI≥24, WC<90 for men and WC<80 for women; BNWO, 18.5≤BMI<24, WC≥90 for men and WC≥80 for women; BOWO, BMI≥24, WC≥90 for men and WC≥80 for women.

**Table 3:** Prevalence of obesity categories by gender and age groups.

Age (years)	N	BNWN*	BOWN*	BNWO*	BOWO*	General obesity	Central obesity
Men							
18–39	813	492 (60.5)	146 (18.0)	6 (0.7)	169 (20.8)	80 (9.8)	175 (21.5)
40–59	2004	1142 (57.0)	400 (20.0)	19 (0.9)	443 (22.1)	162 (8.1)	462 (23.1)
≥60	803	560 (69.7)	115 (14.3)	6 (0.7)	122 (15.2)	26 (3.2)	128 (15.9)
Women							
18–39	1588	819 (51.6)	143 (9.0)	102 (6.4)	524 (33.0)	172 (10.8)	626 (39.4)
40–59	3221	1203 (37.3)	309 (9.6)	257 (8.0)	1452 (45.1)	429 (13.3)	1709 (53.1)
≥60	745	303 (40.7)	38 (5.1)	130 (17.4)	274 (36.8)	79 (10.6)	404 (54.2)

Data are presented as number (percentage).

^*^BNWN, BOWN, BNWO, and BOWO defined using BMI (kg/m2) and WC (cm) levels. BNWN, 18.5≤BMI<24, WC<90 for men and WC<80 for women; BOWN, BMI≥24, WC<90 for men and WC<80 for women; BNWO, 18.5≤BMI<24, WC≥90 for men and WC≥80 for women; BOWO, BMI≥24, WC≥90 for men and WC≥80 for women.

**Table 4:** Cox proportional hazards analysis of association of obesity categories and risk of hypertension by gender and age groups.

Variables	Age 18–39 years		Age 40–59 years		Age ≥60 years	
	HR (95% CI)^a^	HR (95% CI)^b^	HR (95% CI)^a^	HR (95% CI)^b^	HR (95% CI)^a^	HR (95% CI)^b^
Men						
BMI (kg/m^2^) levels						
Normal weight	1.00	1.00	1.00	1.00	1.00	1.00
Overweight	2.13 (1.41–3.22)	1.85 (1.20–2.86)	1.92 (1.54–2.38)	2.15 (1.70–2.71)	1.15 (0.87–1.51)	1.19 (0.85–1.68)
General obesity	4.15 (2.51–6.84)	2.93 (1.68–5.11)	2.13 (1.52–2.99)	2.34 (1.61–3.40)	1.31 (0.64–2.66)	1.24 (0.54–2.83)
WC (cm) levels						
Normal WC	1.00	1.00	1.00	1.00	1.00	1.00
Central obesity	2.61 (1.78–3.84)	1.87 (1.23–2.86)	1.85 (1.49–2.29)	1.83 (1.45–2.31)	1.05 (0.75–1.48)	1.12 (0.75–1.69)
BMI (kg/m^2^) /WC (cm) levels*						
BNWN	1.00	1.00	1.00	1.00	1.00	1.00
BOWN	1.86 (1.14–3.04)	1.78 (1.06–2.99)	1.74 (1.34–2.27)	2.02 (1.53–2.68)	1.27 (0.91–1.78)	1.30 (0.86–1.97)
BNWO	0	0	1.79 (0.73–4.36)	1.12 (0.35–3.53)	2.15 (0.53–8.72)	3.61 (0.84–15.57)
BOWO	3.20 (2.11–4.86)	2.29 (1.45–3.62)	2.20 (1.73–2.79)	2.32 (1.79–3.00)	1.07 (0.75–1.53)	1.13 (0.73–1.73)
Women						
BMI (kg/m^2^) levels						
Normal weight	1.00	1.00	1.00	1.00	1.00	1.00
Overweight	2.25 (1.58–3.19)	1.95 (1.37–2.79)	1.59 (1.33–1.90)	1.57 (1.30–1.89)	1.14 (0.88–1.48)	1.24 (0.90–1.70)
General obesity	4.58 (3.05–6.89)	3.13 (2.02–4.84)	2.08 (1.65–2.62)	2.13 (1.67–2.72)	1.05 (0.70–1.59)	0.99 (0.62–1.59)
WC (cm) levels						
Normal WC	1.00	1.00	1.00	1.00	1.00	1.00
Central obesity	2.89 (2.10–3.98)	2.45 (1.77–3.39)	1.53 (1.30–1.81)	1.50 (1.26–1.79)	0.97 (0.76–1.24)	0.97 (0.72–1.31)
BMI (kg/m^2^) /WC (cm) levels*						
BNWN	1.00	1.00	1.00	1.00	1.00	1.00
BOWN	1.90 (1.03–3.52)	1.53 (0.82–2.84)	1.57 (1.17–2.10)	1.57 (1.14–2.16)	1.10 (0.60–2.00)	1.32 (0.67–2.58)
BNWO	2.18 (1.20–3.96)	1.96 (1.07–3.58)	1.20 (0.87–1.65)	1.14 (0.81–1.60)	0.84 (0.60–1.20)	0.85 (0.55–1.31)
BOWO	3.49 (2.45–4.98)	2.81 (1.96–4.03)	1.81 (1.50–2.18)	1.77 (1.45–2.16)	1.05 (0.80–1.38)	1.08 (0.78–1.51)

Data are expressed as hazard ratios with 95% confidence interval.

BMI, body mass index; WC, waist circumference; HR, hazard ratio; CI, confidence interval.

^*^Based on the diagnose criteria: BNWN, 18.5≤BMI<24, WC<90 for men and WC<80 for women; BOWN, BMI≥24, WC<90 for men and WC<80 for women; BNWO, 18.5≤BMI<24, WC≥90 for men and WC≥80 for women; BOWO, BMI≥24, WC≥90 for men and WC≥80 for women.

^a^Unadjusted model.

^b^Adjusted for age, education level, smoking, drinking, physical activity and family history of hypertension.

## References

[b1] Li D. J. *et al.* Hypertension burden and control in mainland China: Analysis of nationwide data 2003-2012. Int J Cardiol. 184, 637–644 (2015). 25771229 10.1016/j.ijcard.2015.03.045

[b2] He J. *et al.* Premature deaths attributable to blood pressure in China: a prospective cohort study. Lancet. 374, 1765–1772 (2009). 19811816 10.1016/S0140-6736(09)61199-5

[b3] Kotsis V. *et al.* New developments in the pathogenesis of obesity-induced hypertension. J Hypertens. 33,1499–1508 (2015). 26103132 10.1097/HJH.0000000000000645

[b4] Nyamdorj R. *et al.* BMI Compared With Central Obesity Indicators as a Predictor of Diabetes Incidence in Mauritius. Obesity. 17, 342–348 (2009). 19008866 10.1038/oby.2008.503

[b5] Luo W. S. *et al.* Comparison of the Suitability of 2 Years Change in Waist Circumference and Body Mass Index in Predicting Hypertension Risk: A Prospective Study in Chinese-Han. Iran J Public Health. 43, 1212–1220 (2014). 26175975 PMC4500423

[b6] Shuger S. L., Sui X. M., Church T. S., Meriwether R. A. & Blair S. N. Body mass index as a predictor of hypertension incidence among initially healthy normotensive women. Am J Hypertens. 21, 613–619 (2008). 18437123 10.1038/ajh.2008.169 PMC3410431

[b7] Li C., Ford E. S., McGuire L. C. & Mokdad A. H. Increasing trends in waist circumference and abdominal obesity among US adults. Obesity. 15, 216–224 (2007). 17228050 10.1038/oby.2007.505

[b8] Du T. T., Sun X. X., Yin P., Huo R., Ni C. C. & Yu X. F. Increasing trends in central obesity among Chinese adults with normal body mass index, 1993-2009. Bmc Public Health. 13, 327 (2013). 23575244 10.1186/1471-2458-13-327 PMC3626835

[b9] Guo X. *et al.* An update on overweight and obesity in rural Northeast China: from lifestyle risk factors to cardiometabolic comorbidities. Bmc Public Health. 14, 1046 (2014). 25293836 10.1186/1471-2458-14-1046 PMC4198624

[b10] Guo X. F. *et al.* An update on overweight and obesity in rural Northeast China: from lifestyle risk factors to cardiometabolic comorbidities. Bmc Public Health. 14, 1046 (2014). 25293836 10.1186/1471-2458-14-1046 PMC4198624

[b11] Zheng L. *et al.* The association between body mass index and incident hypertension in rural women in China. European journal of clinical nutrition. 64, 769–775 (2010). 20531439 10.1038/ejcn.2010.97

[b12] Wu X. *et al.* Changes in average blood pressure and incidence of high blood pressure 1983-1984 to 1987-1988 in four population cohorts in the People’s Republic of China. The PRC-USA Cardiovascular and Cardiopulmonary Epidemiology Research Group. J Hypertens. 14, 1267–1274 (1996). 8934353 10.1097/00004872-199611000-00003

[b13] Liang Y., Liu R., Du S. & Qiu C. Trends in incidence of hypertension in Chinese adults, 1991-2009: the China Health and Nutrition Survey. Int J Cardiol. 175, 96–101 (2014). 24833472 10.1016/j.ijcard.2014.04.258 PMC4105139

[b14] Jin M. J. *et al.* Prevalence of Overweight and Obesity and Their Associations with Socioeconomic Status in a Rural Han Chinese Adult Population. Plos one 8, e79946 (2013). 24224024 10.1371/journal.pone.0079946 PMC3818265

[b15] Janssen I., Heymsfield S. B., Wang Z. M. & Ross R. Skeletal muscle mass and distribution in 468 men and women aged 18-88 yr. Journal of applied physiology. 89, 81–88 (2000). 10904038 10.1152/jappl.2000.89.1.81

[b16] Huxley R. & Collaboration O. A. Is central obesity a better discriminator of the risk of hypertension than body mass index in ethnically diverse populations? J Hypertens. 26, 169–177 (2008). 18192826 10.1097/HJH.0b013e3282f16ad3

[b17] Dalton M. *et al.* Waist circumference, waist-hip ratio and body mass index and their correlation with cardiovascular disease risk factors in Australian adults. Journal of internal medicine. 254, 555–563 (2003). 14641796 10.1111/j.1365-2796.2003.01229.x

[b18] Chen Z. *et al.* Blood pressure in relation to general and central adiposity among 500 000 adult Chinese men and women. Int J Epidemiol. 44, 1305–1319 (2015). 25747585 10.1093/ije/dyv012 PMC4588860

[b19] Rodriguez B. L., Labarthe D. R., Huang B. & Lopez-Gomez J. Rise of blood pressure with age. New evidence of population differences. Hypertension. 24, 779–785 (1994). 7995637 10.1161/01.hyp.24.6.779

[b20] Sun Z. Q. *et al.* Incidence and Predictors of Hypertension Among Rural Chinese Adults: Results From Liaoning Province. Ann Fam Med. 8, 19–24 (2010). 20065274 10.1370/afm.1018 PMC2807383

[b21] Gu D. F. *et al.* Incidence and predictors of hypertension over 8 years among Chinese men and women. J Hypertens. 25, 517–523 (2007). 17278966 10.1097/HJH.0b013e328013e7f4

[b22] Li Z. *et al.* Association between Ideal Cardiovascular Health Metrics and Depression in Chinese Population: A Cross-sectional Study. Sci Rep. 5, 11564 (2015). 26176196 10.1038/srep11564 PMC4648472

[b23] World Health O. The World Health report 2002. Midwifery. 19, 72–73 (2003). 12691085 10.1054/midw.2002.0343

[b24] Wang C. J. *et al.* Evaluating the risk of type 2 diabetes mellitus using artificial neural network: An effective classification approach. Diabetes Res Clin Pr. 100, 111–118 (2013). 10.1016/j.diabres.2013.01.023 23453177

[b25] Zhang M. *et al.* Development and Validation of a Risk-Score Model for Type 2 Diabetes: A Cohort Study of a Rural Adult Chinese Population. Plos one. 11, e0152054 (2016). 27070555 10.1371/journal.pone.0152054 PMC4829145

[b26] Li Y. Q. *et al.* Resting heart rate as a marker for identifying the risk of undiagnosed type 2 diabetes mellitus: a cross-sectional survey. Bmc Public Health. 14, 1052 (2014). 25297916 10.1186/1471-2458-14-1052 PMC4210587

[b27] Zhao Y. *et al.* Association of obesity categories and high blood pressure in a rural adult Chinese population. J Hum Hypertens. 10, 1038 (2016). 10.1038/jhh.2016.1 26911536

[b28] Geographical variation in the major risk factors of coronary heart disease in men and women aged 35–64 years. The WHO MONICA Project. World health statistics quarterly Rapport trimestriel de statistiques sanitaires mondiales. **41**, 115–140 (1988). 3232405

[b29] Bairaktari E. *et al.* Estimation of LDL cholesterol based on the Friedewald formula and on apo B levels. Clin Biochem. 33, 549–555 (2000). 11124340 10.1016/s0009-9120(00)00162-4

[b30] Chobanian A. V. *et al.* Seventh Report of the Joint National Committee on Prevention, Detection, Evaluation, and Treatment of High Blood Pressure. Hypertension. 42, 1206–1252 (2003). 14656957 10.1161/01.HYP.0000107251.49515.c2

[b31] Zhou B. F. Cooperative Meta-Analysis Group of the Working Group on Obesity in C. Predictive values of body mass index and waist circumference for risk factors of certain related diseases in Chinese adults–study on optimal cut-off points of body mass index and waist circumference in Chinese adults. Biomedical and environmental sciences : BES. 15, 83–96 (2002). 12046553

[b32] Alberti K. G. M. M., Zimmet P. & Shaw J. The metabolic syndrome - a new worldwide definition. Lancet. 366, 1059–1062 (2005). 16182882 10.1016/S0140-6736(05)67402-8

