# Supplementary material for: Body mass index and waist circumference combined predicts obesity-related hypertension better than either alone in a rural Chinese population
Source: Sci Rep. 2016 Aug 22;6:31935. doi: 10.1038/srep31935 (PMC4992958; doi:10.1038/srep31935)
Supplement: Supplementary Information [file srep31935-s1.pdf]

Title:

Body mass index and waist circumference combined predicts obesity-related hypertension better than either alone in a rural Chinese population

Authors:

Ming Zhang, Yang Zhao, Guoan Wang, Hongyan Zhang, Yongcheng Ren, Bingyuan Wang, Lu Zhang, Xiangyu Yang, Chengyi Han, Chao Pang, Lei Yin, Jingzhi Zhao, Dongsheng Hu

Supplementary Table 1. Awareness, treatment, and control of hypertension in study population

Variables	Awareness	Treatment	Control
**Overall**	865 (48.5)	840 (47.1)	475 (26.6)
**Gender**			
**Men**	350 (47.7)	323 (44.1)	180 (24.6)
**Women**	515 (49.0)	517 (49.2)	295 (28.1)
**Age (years)**			
**18-39**	109 (39.2)	93 (33.5)	55 (19.8)
**40-59**	503 (50.8)	494 (49.9)	304 (30.7)
**>60**	253 (49.0)	253 (49.0)	116 (22.5)

Data are number (percentage).
